# ST8SIA6 Sialylates CD24 to Enhance Its Membrane Localization in BRCA

**DOI:** 10.3390/cells14010009

**Published:** 2024-12-26

**Authors:** Jinxia He, Fengchao Zhang, Baihai Wu, Wengong Yu

**Affiliations:** 1 Key Laboratory of Marine Drugs (Ministry of Education), Shandong Provincial Key Laboratory of Glycoscience and Glycoengineering, School of Medicine and Pharmacy, Ocean University of China, Qingdao 266003, China; jxhe2016@163.com (J.H.); zhangfengchaoouc@163.com (F.Z.); wubaihaiouc@163.com (B.W.); 2 Laboratory for Marine Drugs and Bioproducts, Qingdao Marine Science and Technology Center, Qingdao 266237, China

**Keywords:** CD24, subcellular localization, ST8SIA6, sialylation

## Abstract

CD24, a highly sialylated glycosyl-phosphatidyl-inositol (GPI) cell surface protein that interacts with sialic acid-binding immunoglobulin-like lectins (Siglecs), serves as an innate immune checkpoint and plays a crucial role in inflammatory diseases and tumor progression. Recently, cytoplasmic CD24 has been observed in samples from patients with cancer. However, whether sialylation governs the subcellular localization of CD24 in cancer remains unclear, and the impact of CD24 expression and localization on the clinical prognosis of cancer remains controversial. Here, we performed a systematic pan-cancer analysis of the gene expression levels and clinical correlation of *CD24*. Our analysis revealed that *CD24* was highly expressed in breast tumor tissues and tumor cells, significantly shortening patient survival time. However, this correlation was not evident in other types of cancer. Additionally, a correlation analysis of CD24 levels with sialyltransferases (STs) revealed that ST8SIA6 is the key ST affecting CD24 sialylation. Further investigation demonstrated that ST8SIA6 directly modified CD24, promoting its localization to the cell membrane. Taken together, these findings elucidate, for the first time, the mechanisms by which ST8SIA6 regulates CD24 subcellular localization, providing new insights into the biological functions and applications of CD24.

## 1. Introduction

CD24 is a glycosyl-phosphatidyl-inositol-anchored (GPI) surface protein [1,2,3]. It is upregulated in human malignancies, such as breast cancer and ovarian cancer, and serves as a prognostic marker [4,5,6,7,8,9]. However, an increasing number of studies have observed cytoplasmic accumulation of CD24 in invasive and metastatic cancers, such as ovarian cancer [6,7], colorectal cancer [10,11], and breast cancer [4]. This phenomenon may be due to the widespread disruption of genes in processing GPI-anchored molecules [12,13]. Currently, the impact of *CD24* expression on patients’ prognosis and tumor progression remains the subject of debate. For instance, in colorectal cancer, some studies have indicated that high levels of *CD24* significantly shorten patient survival time [10,14], whereas another study showed that high levels of *CD24* were associated with prolonged patient survival [11]. The expression of CD24 promotes the progression and metastasis of prostate cancer [13], but inhibits the invasiveness and metastasis of pancreatic cancer [15]. Additionally, the effect of subcellular localization on clinical prognosis remains controversial. For example, in ovarian [6] and gastric cancers [16], the expression of cytoplasmic CD24 significantly reduces the overall survival rate of patients. In non-small cell lung cancer, the expression of the membrane CD24 is associated with lymphonodular spread and shorter overall survival times [17]. A recent study demonstrated that the translocation of CD24 from the cytosol to the cell surface triggers treatment resistance [18]. However, the regulatory mechanisms controlling the subcellular localization of CD24 in cancer remain unclear.

Cell surface CD24 exhibits high levels of sialylation [1,2]. Owing to the heterogeneity of sialoglycans, it is not surprising that CD24 mediates different functions based on its interaction with various sialic-acid-binding immunoglobulin-like lectin (Siglec) receptors [19]. For instance, in metaflammation, sialylated CD24 interacts with Siglec-E (homolog of human Siglec-7/9) to suppress the production of inflammatory cytokines and protect against metabolic disorders [20,21]. Conversely, in ovarian cancer and triple-negative breast cancer (TNBC), sialylated CD24 on the surface of tumor cells serves as a “don’t eat me” signal by binding to Siglec-10 on macrophages, thereby preventing the clearance of cancer cells and facilitating tumor progression [3]. Overall, the mechanism of membranous CD24 as an innate immune checkpoint interacting with Siglecs is well understood. However, the key sialyltransferases (STs) that directly modify CD24 have not been identified.

Currently, the 20 known STs are mainly categorized into α2,3 (ST3), α2,6 (ST6), and α2,8 (ST8), based on the donor sialic acid attachment location on the receptor [22,23]. The ST3 and ST6 families are primarily responsible for adding the first SA to the sugar structure of glycoproteins or glycolipids, and the subsequent extension of the sugar structure requires the ST8 family. In other words, once the ST8 family adds SA to the end of the sugar chain, other types of sugar residues can no longer continue sugar chain derivation. Thus, the ST8 family is the last step in sugar chain sialylation. To date, six members of the ST8 family, namely ST8SIA1–6, have been identified in both mice and humans [24,25].

The aim of this study was to comprehensively and systematically perform a pan-cancer analysis of the expression of *CD24* and its impact on cancer prognosis, further identifying the key α2,8-STs that affect the terminal sialylation of CD24, as well as revealing the regulatory mechanisms governing the subcellular localization of CD24 in cancer.

## 2. Materials and Methods

### 2.1. Cell Culture

The 293T cell line, originally named 293tsA1609neo, is a highly transfection-efficient derivative obtained by inserting the SV40 T-antigen gene into the human embryonic kidney cell line 293 [26]. The 4T1 cell line, a thioguanine-resistant variant selected from 410.4 without mutagen treatment, exhibits tumor growth and metastatic spread in BALB/c mice similar to those in human BRCA [27,28]. 293T and 4T1 cell lines were obtained from the Cell Bank/Stem Cell Bank, Chinese Academy of Sciences, and maintained according to the manufacturer’s instructions. 293T cells were cultured in Dulbecco’s Modified Eagle Medium (Gibco, Waltham, MA, USA, Cat.# 12800-017) with 10% fetal bovine serum (FBS, Limassol, Cyprus; PAN-Biotech, Aidenbach, Germany, Cat.# ST30-3302) under 5% CO_2_ at 37 °C. 4T1 cells were cultured in RPMI-1640 (Gibco, Waltham, MA, USA, Cat.# 31800-022) medium supplemented with 10% FBS under 5% CO_2_ at 37 °C.

### 2.2. Dataset Analysis

**Figure 1:**
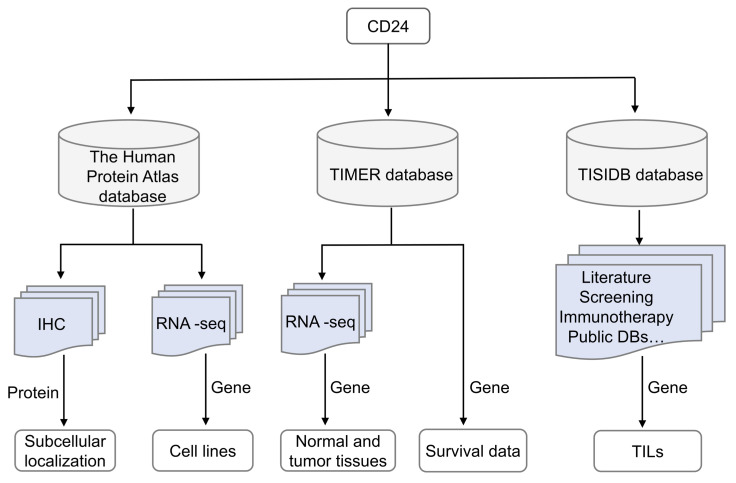
The flowchart illustrates the pan-cancer analysis of CD24. Abbreviations: IHC, immunohistochemistry; RNA-seq, RNA sequencing; DBs, databases; TILs, tumor-infiltrating lymphocytes.

Figure 1 illustrates the pan-cancer analysis process of CD24. The TIMER database (http://timer.cistrome.org/) (accessed on 1 March 2023) was employed to estimate *CD24* gene expression levels in human cancers [29,30]. The Human Protein Atlas database (https://www.proteinatlas.org/) (accessed on 1 March 2023) was utilized to assess the gene expression levels of *CD24* in various human cancer cell lines and to analyze the subcellular localization of the CD24 protein. The TISIDB database (http://cis.hku.hk/TISIDB/) (accessed on 1 March 2023) was used to determine the correlation between *CD24* expression and the abundance of tumor-infiltrating lymphocytes (TILs), including activated CD8^+^ T cells, activated CD4^+^ T cells, natural killer cells, activated dendritic cells, macrophages, monocytes, and neutrophils [31]. The correlations between *CD24* levels and ST8 family member levels, as well as the correlations between *Siglec-7/9/10* levels and tumor-infiltrating macrophage abundance in BRCA, were analyzed using the TIMER database. Heatmaps were generated using TBtools (version 2.127) software [32].

### 2.3. Plasmid Construction

A lentiviral vector containing the complete coding sequence of murine *St8sia6* (pLVEXP01-puro-m*St8sia6*) was obtained from Cyagen Biosciences (Suzhou, China). The nontargeting vector pLVEXP01-puro-empty was used as a control. *St8sia6*-knockout constructs were generated using a lentiCRISPR v2-puro plasmid (Addgene, Watertown, MA, USA, Cat.# 52961). Single-guide RNAs were designed using an online tool (http://crispor.tefor.net/crispor.py) (accessed on 3 March 2022). An efficient CRISPR target sequence was identified in exon 1 (CTGGGCACCAGAGCATACGC). Guide RNAs were synthesized in vitro using a PCR product-based approach as a target guide sequence cloning protocol, as reported by the Zhang Feng Lab [33]. The inserts were sequenced to ensure the absence of unintended mutations. The endotoxin-free plasmid was obtained using the E.Z.N.A. Enda-Free Plasmid Mini Kit II (OMEGA, Mumbai, India, Cat.# D6950-01).

### 2.4. Lentiviruses Production and Generation of Stable Cell Lines

Lentiviruses were produced using the Addgene protocol (https://www.addgene.org/protocols/lentivirus-production/) (accessed on 3 March 2022). Specifically, 5 million cells were seeded in 6 cm tissue culture dishes the day before transfection. The plasmid and virus packaging vectors psPAX2 (Addgene, Watertown, MA, USA, Cat.# 12260), pMD2.G (Addgene, Watertown, MA, USA, Cat.# 12259), and PEI (Sigma-Aldrich, Saint Louis, MO, USA, Cat.# 795090) were separately diluted with Opti-MEM (Gibco, Waltham, MA, USA, Cat.# 51985034). After gently mixing the diluted plasmid and PEI (at a ratio of 1 μg of total DNA to 3 μg of PEI), followed by a 15-min incubation, the mixture was added dropwise to 293T cells. The medium was changed 6 h after transfection. Two days after the initial transfection, the virus-containing supernatant was collected and filtered through a 0.45 μm syringe filter. The virus-containing supernatant was collected for a second time 72 h after transfection. A total of 2 × 10^5^ 4T1 cells/well were seeded in a 6-well plate. On the following day, the medium was replaced with the harvested virus. After 24 h of transduction, the medium was replaced with a mixture of 50% virus-containing medium and 50% fresh medium, which was maintained for an additional 24 h. Subsequently, 48 h after the initial transduction, the medium was replaced with a normal culture medium. *St8sia6*-overexpressing (OE-*St8sia6*) and *St8sia6*-knockout (sg*St8sia6*) cells were selected by treatment with 0.25 μg/mL puromycin (Solarbio, Beijing, China, Cat.# 58-58-2) for 3 days.

### 2.5. Tumor Models

Female BALB/c mice aged 6–8 weeks were obtained from Beijing Vital River Laboratory Animal Technology Co., Ltd., Beijing, China for use in all in vivo experiments. The experimental procedures were conducted according to the protocols approved by the Ethics and Animal Welfare Committee of the School of Medicine and Pharmacy at the Ocean University of China. To establish a tumorigenicity model, 1 × 10^6^ 4T1 tumor cells were suspended in a solution containing a mixture of Matrigel (Corning, New York, NY, USA, Cat.# 354248) and phosphate-buffered saline (PBS). This solution was then injected into the 4th right mammary fat pad of the BALB/c mice. After 30 days, the mice were euthanized, and the tumors were harvested for further analysis and experiments.

### 2.6. Flow Cytometry

Cells were fixed in 4% paraformaldehyde for 10 min, washed with PBS, and then incubated with Alexa-Fluor^®^-488-conjugated anti-mouse-CD24 antibodies (0.5 μg/10^6^ cells; R&D Systems, Minneapolis, MN, USA; Cat.# FAB8547G) for 30 min at 4 °C. Subsequently, the cells were washed with PBS, resuspended in isotonic buffer, and filtered through a 200-mesh filter for further analysis of membrane protein expression using flow cytometry (NovoCyte, Agilent, Santa Clara, CA, USA). To detect total protein, the cells were permeabilized with PBS containing 1% Triton X-100 (*v*/*v*) for 20 min after fixation with paraformaldehyde to allow for antibody access to the intracellular compartments.

### 2.7. Western Blotting

Cells or tumor tissues were lysed by ultrasonication in RIPA buffer (50 mM Tris-HCl [pH 7.4], 150 mM NaCl, 1% NP40, 0.5% sodium deoxycholate, 0.1% SDS, 1 mM EDTA, 1 mM Na3VO4, and 10 mM NaF supplemented with PMSF and complete protease inhibitors) for 6 min in an ice bath. The lysates were centrifuged for 15 min at 12,000 rpm and 4 °C. The cleared lysates were collected, and the protein concentration was determined using a bicinchoninic acid protein assay kit (New Cell & Molecular Biotech Co., Ltd., Newcastle Upon Tyne, UK, Cat.# WB6501). The soluble fraction was subjected to electrophoresis on a gradient gel (4–12% SDS-PAGE), transferred onto an Immobilon-FL PVDF membrane (Millipore, Singapore, Cat.# IPVH00010), and immunoblotted with the indicated antibodies. The following antibodies were used for immunoblotting analysis: anti-ST8SIA6 (1:1000, Sigma-Aldrich, Cat.# SAB2105579), anti-β-actin (1:1000, Cell Signaling Technology, Danvers, MA, USA, Cat.# 3700S), HRP AffiniPure™ goat anti-rabbit IgG Fc (1:10,000, Millipore, Cat.# AP156P) and horseradish-peroxidase-conjugated AffiniPure™ goat anti-mouse IgG (1:10,000, Jackson ImmunoResearch, West Grove, PA, USA, Cat.# 115-035-003).

### 2.8. Immunofluorescence

Immunofluorescence analysis of cells was performed as described previously [34]. Briefly, the cells were fixed in 4% paraformaldehyde for 30 min, washed with PBS, and permeabilized with PBS containing 0.5% (*v*/*v*) Triton X-100 for 5 min. The cells were then washed with PBS, incubated with the indicated primary antibodies overnight at 4 °C, washed with PBS again, and incubated with the appropriate secondary antibodies for 60 min at room temperature. Images were acquired using a Leica TCS SP8 STED microscope with a silicon immersion 40× or 63× objective lens. The following fluorescent antibodies were used: anti-CD24 (1:200, Invitrogen, Cat.# 14-0242-82), anti-ST8SIA6 (1:200, Creative Biolabs, Shirley, NY, USA, Cat.# CBMAB-S4944-CQ), anti-Siglec-E (1:200, R&D Systems, Cat.# AF5806), recombinant mouse Siglec-E Fc (1:100, R&D Systems, Cat.# 5806-SL), Alexa-Fluor-594-conjugated goat anti-mouse IgG (1:400, Abcam, Cambridge, UK, Cat.# ab150116), and FITC-conjugated goat anti-rat IgG (1:400, Servicebio, Wuhan, China, Cat.# GB22302). Tissue specimens were prepared and stained by Service Bio (Wuhan, China).

### 2.9. Statistical Analysis

Data are presented as the mean ± standard error of the mean. Statistical significance was determined using one-way analysis of variance (ANOVA) with SPSS 17.0. The level of significance was indicated as * *p* < 0.05, ** *p* < 0.01, and *** *p* < 0.001.

## 3. Results

### 3.1. Pan-Cancer Correlation Analysis of CD24 Expression Level and Prognosis

**Figure 2:**
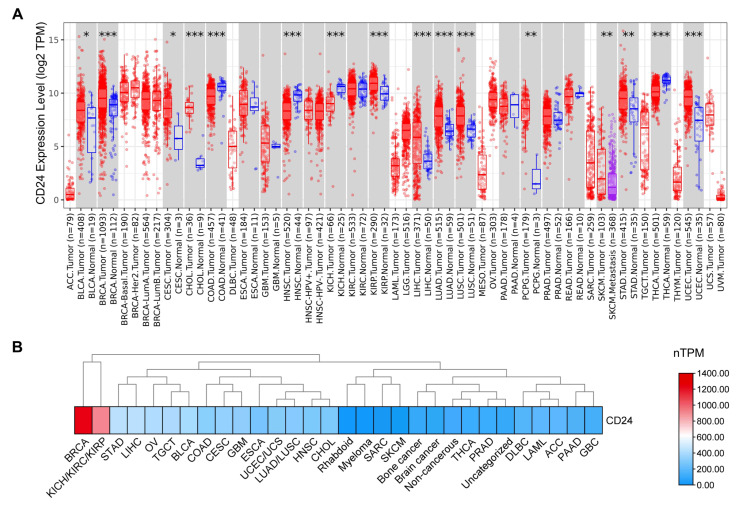
Expression analysis of the *CD24* gene in various human cancers. (**A**) The RNA expression levels of *CD24* in human cancers were analyzed using the TIMER algorithm. The statistical significance computed by the Wilcoxon test is annotated by the number of asterisks. * *p* < 0.05, ** *p* < 0.01, *** *p* < 0.001. (**B**) The RNA expression levels of *CD24* in various human cancer cell lines were analyzed using data from the Human Protein Atlas database.

**Figure 3:**
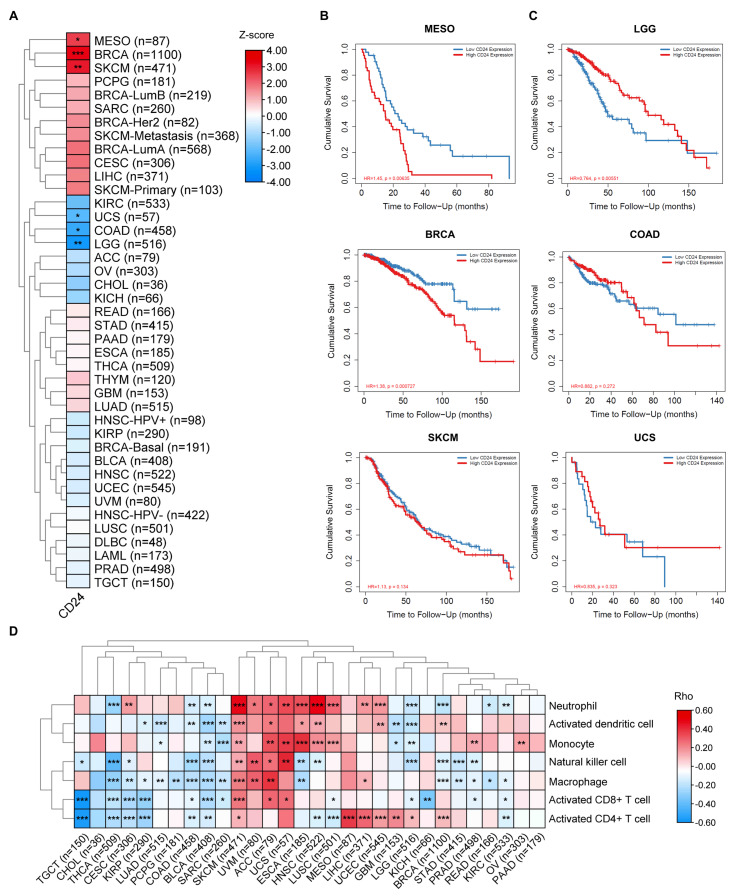
The clinical relevance of *CD24* gene expression levels across various cancer types. (**A**) The clinical correlation between *CD24* gene expression levels and various types of cancer was analyzed using the TIMER algorithm, incorporating key clinical factors, including age, tumor stage, and tumor purity. A heatmap was drawn to show the normalized coefficient of the gene in the Cox model. Z-Score > 0, *p* < 0.05, increased risk; Z-Score < 0, *p* < 0.05, decreased risk; *p* > 0.05, not significant. * *p* < 0.05, ** *p* < 0.01, *** *p* < 0.001. (**B**) The impact of *CD24* levels on the survival rates of patients with BRCA, MESO, and SKCM. HR represents the hazard ratio. (**C**) The impact of *CD24* levels on the survival rates of patients COAD, LGG, and UCS. HR represents the hazard ratio. (**D**) Spearman’s correlations between *CD24* gene expression levels and TILs, including activated CD8^+^ T cells, activated CD4^+^ T cells, natural killer cells, activated dendritic cells, macrophages, monocytes, and neutrophils, across various types of human cancers were obtained from the TISIDB database. Rho represents the Spearman’s correlation coefficient. Spearman’s rho > 0, *p* < 0.05, positive correlation; Spearman’s rho < 0, *p* < 0.05, negative correlation; *p* > 0.05, not significant. * *p* < 0.05, ** *p* < 0.01, *** *p* < 0.001.

First, *CD24* gene expression levels in human cancers were analyzed using the TIMER database (http://timer.cistrome.org/) (accessed on 1 March 2023) [29,30]. The data showed that *CD24* expression was significantly upregulated in most cancers, including breast carcinoma (BRCA), cholangiocarcinoma (CHOL), kidney renal papillary cell carcinoma (KIRP), liver hepatocellular carcinoma (LIHC), lung adenocarcinoma (LUAD), lung squamous carcinoma (LUSC), uterine corpus endometrial carcinoma (UCEC), pheochromocytoma and paraganglioma (PCPG), stomach adenocarcinoma (STAD), bladder urothelial carcinoma (BLCA), and cervical squamous carcinoma (CESC) (Figure 2A). However, *CD24* expression was significantly downregulated in colon adenocarcinoma (COAD), head and neck squamous cell carcinoma (HNSC), kidney renal clear cell carcinoma (KIRC), and thyroid carcinoma (THCA) (Figure 2A). We examined the gene expression levels of *CD24* in various human cancer cell lines using data from the Human Protein Atlas database (https://www.proteinatlas.org/) (accessed on 1 March 2023). The results showed that *CD24* was expressed in all cancer cell lines, with the highest levels observed in BRCA cell lines (Figure 2B). To understand the impact of *CD24* on patient prognosis, we analyzed the clinical correlation between *CD24* expression levels and various cancer types based on key clinical factors, including age, tumor stage, and tumor purity. The results showed that *CD24* significantly increased the risk of tumorigenesis and the progression of BRCA, mesothelioma (MESO), and skin cutaneous melanoma (SKCM) (Figure 3A). Moreover, high expression levels of *CD24* significantly shortened the overall survival time of patients with BRCA and MESO (Figure 3B). Conversely, *CD24* significantly reduced the disease risk of COAD, lower grade glioma (LGG), and uterine carcinosarcoma (UCS) (Figure 3A). High expression levels of CD24 significantly prolonged the overall survival time of patients with LGG (Figure 3C).

Next, we analyzed the correlation between *CD24* expression level and the abundance of tumor-infiltrating lymphocytes (TILs), including activated CD8^+^ T cells, activated CD4^+^ T cells, natural killer cells, activated dendritic cells, macrophages, monocytes, and neutrophils, using the TISIDB database (http://cis.hku.hk/TISIDB/) (accessed on 1 March 2023) [31]. A positive correlation was observed between *CD24* expression levels and the infiltration of activated CD8^+^ T cells, activated CD4^+^ T cells, natural killer cells, activated dendritic cells, macrophages, monocytes, and neutrophils in SKCM, uveal melanoma (UVM), adrenocortical carcinoma (ACC), and UCS (Figure 3D). However, a negative correlation was observed between *CD24* expression levels and the abundance of TILs in COAD, BLCA, and sarcoma (SARC) (Figure 3D). Notably, the expression level of *CD24* was positively correlated with CD4^+^ T cell activation in MESO, LIHC, UCEC, glioblastoma multiform (GBM), LGG, and BRCA (Figure 3D). The expression level of *CD24* was negatively correlated with the activation of CD4^+^ and CD8^+^ T cells in testicular germ cell tumors (TGCT), THCA, CESC, and KIRP (Figure 3D).

**Figure 4:**
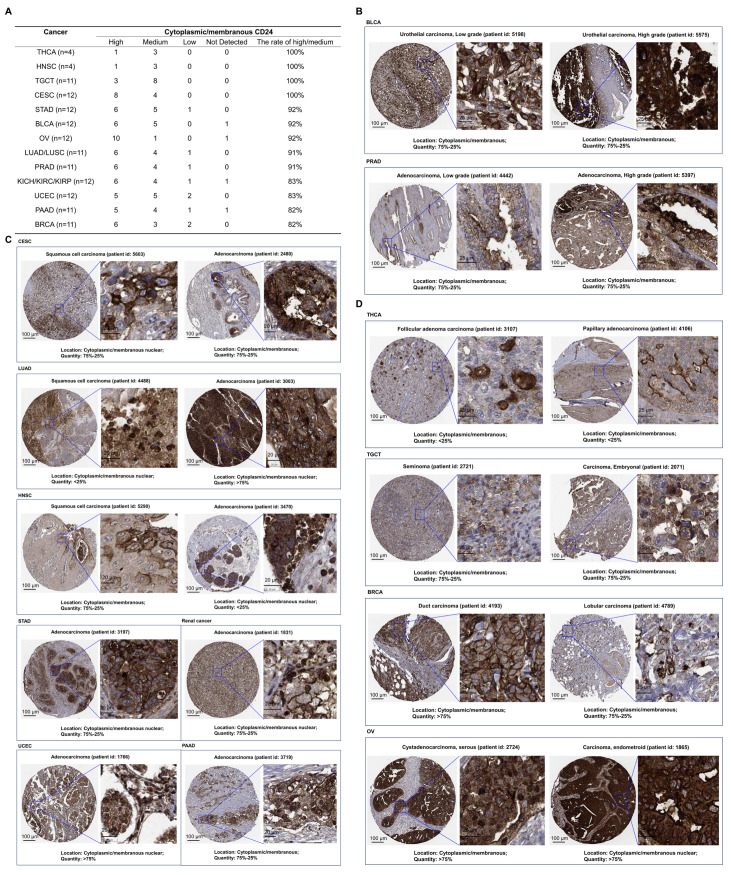
The subcellular localization of CD24 protein was analyzed using data from the Human Protein Atlas database. (**A**) The 13 cancer tissues with the highest levels of CD24 in the cytoplasm and membrane, namely, THCA, HNSC, TGCT, CESC, STAD, BLCA, OV, lung cancer (LUAD and LUSC), PRAD, renal cancer (KICH, KIRC, and KIRP), UCEC, PAAD, and BRCA. (**B**) Representative immunohistochemical staining images of changes in membranous and cytoplasmic CD24 levels in high- and low-stage tumors from BLCA and PARD patients are shown (scale bar, 100 μm and 25 μm). (**C**) Representative immunohistochemical staining images of CD24 in the nucleus (scale bar, 100 μm and 20 μm). (**D**) Representative immunohistochemical staining images of membranous and cytoplasmic CD24 in THCA, TGCT, OV, and BRCA (scale bar, 100 μm and 25 μm).

Finally, we analyzed the subcellular localization of CD24 using data from the Human Protein Atlas database. CD24 expression was detected in the membrane and cytoplasm of most cancers, including THCA, HNSC, TGCT, CESC, STAD, BLCA, ovarian serous cystadenocarcinoma (OV), LUAD, LUSC, prostate adenocarcinoma (PRAD), kidney chromophobe (KICH), KIRC, KIRP, UCEC, PAAD, BRCA, lymphoma, GBM, skin cancer, COAD, LIHC, carcinoids, and SKCM (Figure 4). In 65% of cancers, such as THCA and BRCA, high levels of CD24 were present in the cytoplasm and cell membrane in more than 80% of cases (Figure 4A). Furthermore, the levels of CD24 in the cytoplasm and membrane were significantly enhanced in high-stage tumors compared to low-stage tumors, including BLCA and PRAD (Figure 4B). Moreover, we observed the localization of CD24 in the nucleus of cancers, such as CESC, LUAD, HNSC, and STAD (Figure 4C). In addition, CD24 was observed in the cytoplasm and membrane in common clinical classifications of cancer, such as follicular adenoma carcinoma and papillary adenocarcinoma (THCA); seminoma and embryonal carcinoma (TGCT); duct carcinoma and lobular carcinoma (BRCA); and cystadenocarcinoma, serous carcinoma, and endometrial carcinoma (OV) (Figure 4D).

Altogether, bioinformatics analysis revealed that *CD24* is upregulated in most cancers and is widely distributed in the membrane, cytoplasm, and nucleus. Furthermore, we found a significant negative correlation between *CD24* expression level and BRCA prognosis. However, this correlation was not significant in other cancers. Therefore, the subsequent investigation focused on BRCA.

### 3.2. CD24 Expression Levels Were Significantly Correlated with ST8SIA6 Levels in BRCA

**Figure 5:**
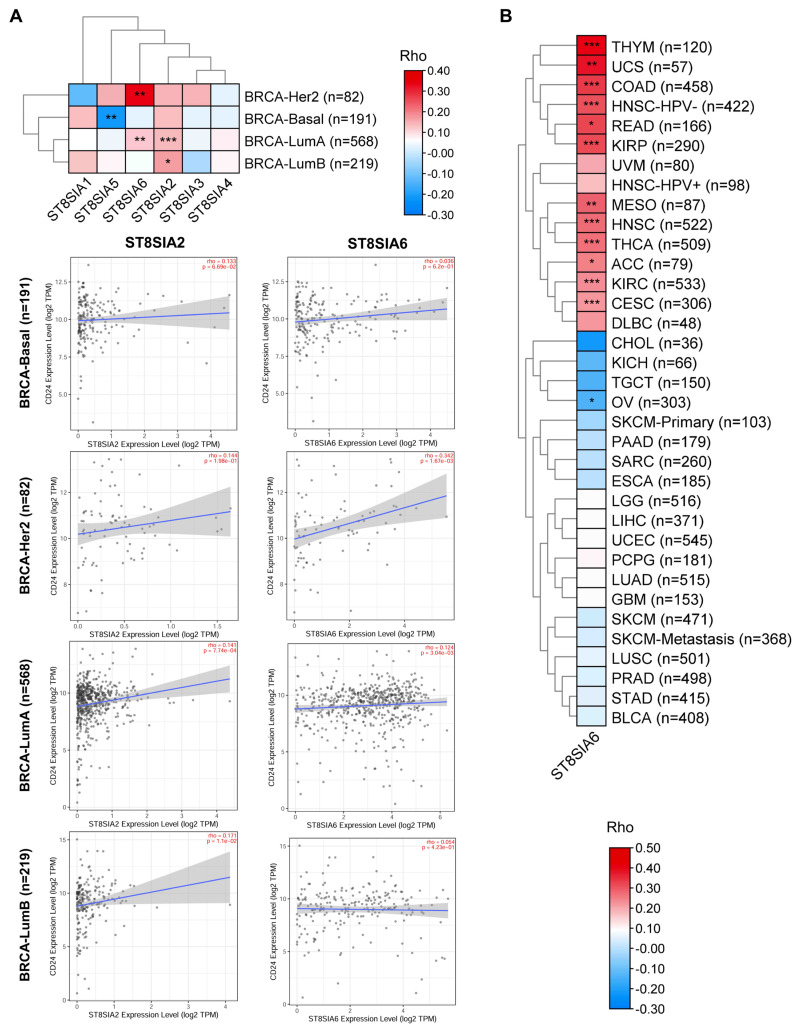
The correlations between *CD24* levels and the levels of ST8 family members in human cancer were analyzed using the TIMER database. (**A**) *CD24* levels were positively correlated with *ST8SIA2* and *ST8SIA6* levels in BRCA. Spearman’s rho > 0, *p* < 0.05, positive correlation; Spearman’s rho < 0, *p* < 0.05, negative correlation; *p* > 0.05, not significant. * *p* < 0.05, ** *p* < 0.01, *** *p* < 0.001. (**B**) The correlations between *CD24* and *ST8SIA6* levels were analyzed in various human cancers. Spearman’s rho > 0, *p* < 0.05, positive correlation; Spearman’s rho < 0, *p* < 0.05, negative correlation; *p* > 0.05, not significant. * *p* < 0.05, ** *p* < 0.01, *** *p* < 0.001.

Aberrant sialylation has been identified as a marker of malignancy, with CD24 showing high levels of sialylation [1,2,35]. Given that the terminality of the ST8 family is responsible for sialylation, we analyzed the correlation between *CD24* expression level and the levels of members of the ST8 family in BRCA using the TIMER algorithm. The results showed that *ST8SIA2* and *ST8SIA6* levels were significantly positively correlated with *CD24* expression levels in BRCA (Figure 5A). Since ST8SIA2 is primarily responsible for the formation of polysialic acid on neural cell adhesion molecules (NCAMs) to participate in brain development [36,37], we speculated that ST8SIA6 may play a crucial role in modifying CD24 in BRCA. Furthermore, we found that *ST8SIA6* levels were positively correlated with *CD24* levels in several cancers, including ACC, COAD, HNSC, rectal adenocarcinoma (READ), THCA, KIRP, thymoma (THYM), UCS, CESC, KIRC, and MESO (Figure 5B).

**Figure 6:**
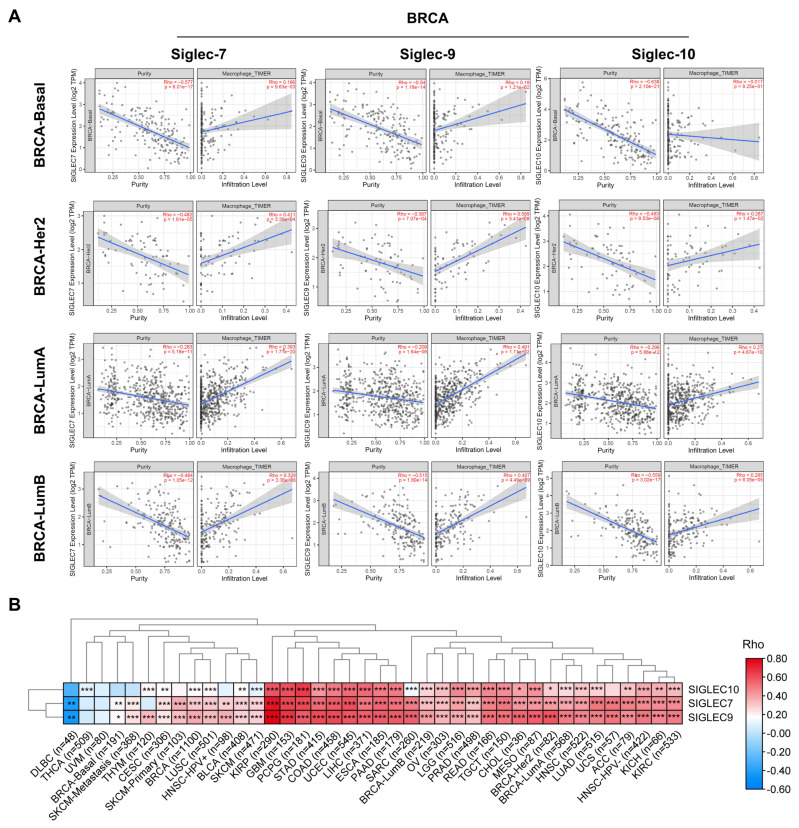
The correlations between *Siglec-7/9/10* levels and the abundance of tumor-infiltrating macrophages in BRCA (**A**) and other human cancers (**B**) were analyzed using the TIMER database. Spearman’s rho > 0, *p* < 0.05, positive correlation; Spearman’s rho < 0, *p* < 0.05, negative correlation; *p* > 0.05, not significant. * *p* < 0.05, ** *p* < 0.01, *** *p* < 0.001.

The interaction between sialylated CD24 on tumor cells and inhibitory Siglecs on macrophages forms the CD24-Siglecs axis, which serves as a glycol-immune checkpoint, enhancing immunosuppression and promoting tumor growth [3]. Therefore, we analyzed the correlation between Siglec receptor expression levels and macrophage infiltration in BRCA. The results showed that the expression level of *Siglec-7/9/10* was significantly positively correlated with macrophage infiltration (Figure 6A). Furthermore, *Siglec-7/9/10* levels were positively associated with macrophage infiltration in 90% of cancers (Figure 6B). These results emphasize the critical role of the terminal sialylation of ST8SIA6 in mediating the interaction between CD24 and Siglecs on macrophages.

### 3.3. CD24 Was Directly Modified by ST8SIA6

We hypothesized that CD24 was directly modified by ST8SIA6. The NeuAcα2,8NeuAc structure catalyzed by ST8SIA6 is the ligand for murine Siglec-E [38,39]. Thus, the NeuAcα2,8NeuAc structure of CD24 can be detected using Siglec-E Fc.

**Figure 7:**
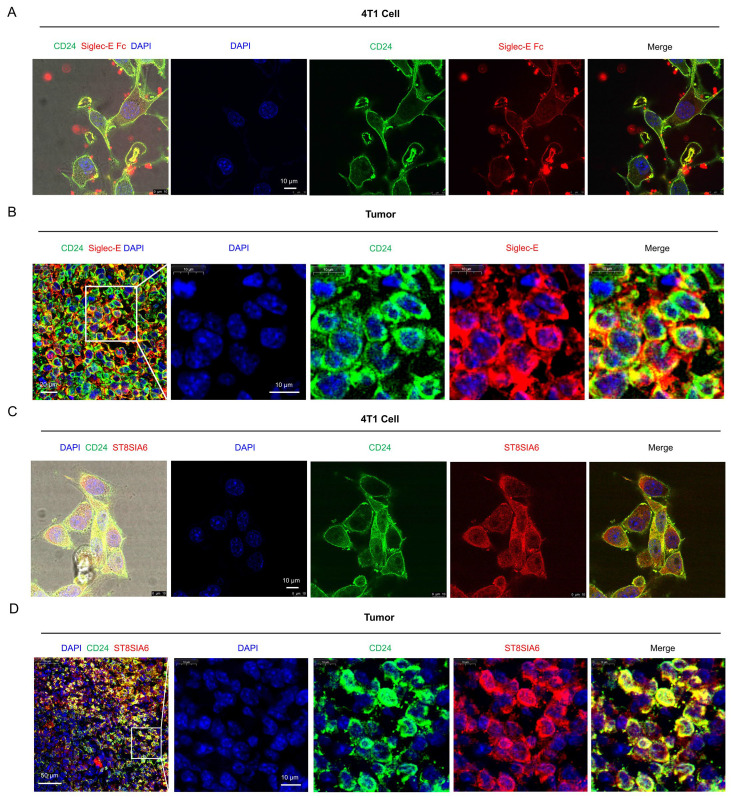
CD24 is directly modified by ST8SIA6. (**A**) The co-localization of CD24 and Siglec-E Fc fluorescence was observed in 4T1 cells (scale bar, 10 μm). (**B**) The co-localization of CD24 and Siglec-E fluorescence was observed in TNBC tumor tissues (scale bar, 20 μm and 10 μm). (**C**) The co-localization of CD24 and ST8SIA6 fluorescence was observed in 4T1 cells (scale bar, 10 μm). (**D**) The co-localization of CD24 and ST8SIA6 fluorescence was observed in TNBC tumor tissues (scale bar, 50 μm and 10 μm).

First, we used a commercial recombinant Siglec-E Fc protein to detect its binding to CD24 on the surface of 4T1 cells by immunofluorescence. Fluorescent co-localization of CD24 and Siglec-E Fc was observed in 4T1 cells (Figure 7A), demonstrating the binding between Siglec-E Fc and CD24. Meanwhile, we detected the binding of endogenous Siglec-E to CD24 in 4T1 tumor tissues by fluorescence immunohistochemistry using anti-Siglec-E antibodies (Figure 7B). These results indicate the existence of the NeuAcα2,8NeuAc structure on the surface of CD24.

Next, we detected the interaction between CD24 and ST8SIA6 in 4T1 cells and BRCA tumor tissues using immunofluorescence co-localization. The results showed that the co-localization of ST8SIA6 and CD24 occurred in 4T1 cells (Figure 7C) and BRCA tumor tissues (Figure 7D). Additionally, we detected an interaction between CD24 and ST8SIA6 through immunoprecipitation (Supplementary Scheme S1). Taken together, these results demonstrated that CD24 is directly modified by ST8SIA6.

### 3.4. ST8SIA6 Promoted the Membrane Localization of CD24

**Figure 8:**
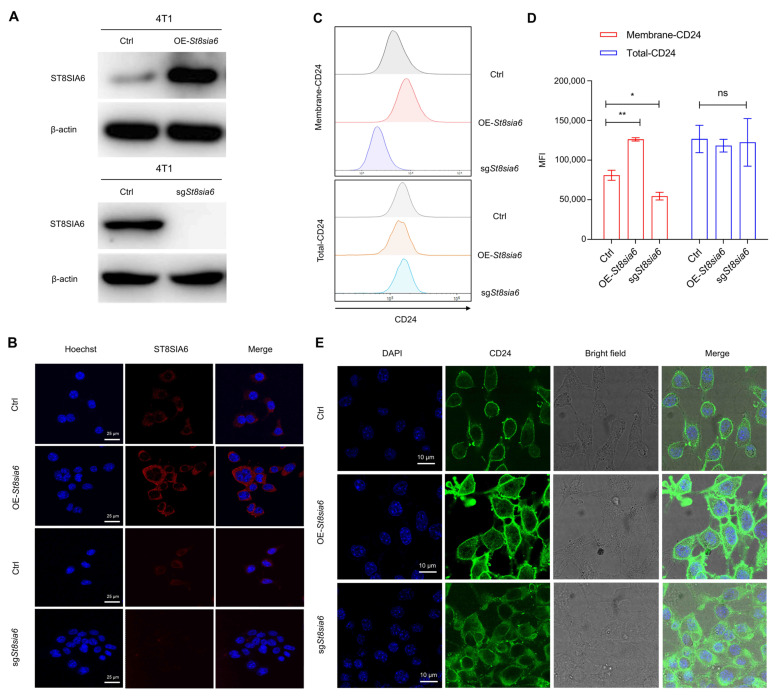
The impact of ST8SIA6 on the subcellular localization and expression of CD24. (**A**) The expression of ST8SIA6 was detected by western blotting in *St8sia6*-overexpressing (OE-*St8sia6*) and *St8sia6*-knockout (sg*St8sia6*) 4T1 cells. (**B**) The expression of ST8SIA6 was detected by immunofluorescence in OE-*St8sia6* and sg*St8sia6* 4T1 cells (scale bar, 25 μm). (**C**) The effects of ST8SIA6 on CD24 expression were detected by flow cytometry. (**D**) Quantitative analysis of CD24 expression in 4T1 cells. The graph shows the median fluorescence intensity (MFI) of CD24. Data are presented as the mean ± standard error of the mean. The *p*-values were calculated using a one-way analysis of variance. * *p* < 0.05, ** *p* < 0.01. (**E**) The effect of ST8SIA6 on the subcellular localization of CD24 was determined by immunofluorescence (scale bar, 10 μm).

To reveal the effect of ST8SIA6 on the expression and localization of CD24, we generated 4T1 cell lines with *St8sia6* overexpression (OE-*St8sia6*) and knockout (sg*St8sia6*) (Figure 8A,B). Initially, the effects of ST8SIA6 on CD24 expression were detected using flow cytometry. The results showed that ST8SIA6 significantly promoted the expression of CD24 on the cell membrane (Figure 8C,D). However, the overall CD24 protein expression level remained unaffected (Figure 8C,D).

Immunofluorescence analysis was performed to assess the effect of ST8SIA6 on the localization of CD24. The results showed that ST8SIA6 significantly enhanced the membrane localization of CD24, and these results were consistent with the flow cytometry results (Figure 8E). These results suggested that CD24 was directly modified by ST8SIA6, and this modification can promote the localization of the CD24 protein on the cell membrane.

## 4. Discussion

CD24 acts as a prognostic marker or innate immune checkpoint and plays a crucial role in the progression of multiple diseases, including inflammation, autoimmune disorders, and tumors [8,40]. In this study, we discovered, for the first time, that CD24 is directly modified by ST8SIA6. Furthermore, this modification promoted the localization of CD24 to the cell membrane.

CD24 has been found to be overexpressed in approximately 70% of human cancers [13,41]. Its effect on patient prognosis varies among different types of cancer [6,13,15,16,17] and within the same type [10,11,14]. In this study, we performed a systematic pan-cancer analysis of the *CD24* gene expression and clinical correlation. Based on the bioinformatics data, we found a significant negative correlation between *CD24* levels and BRCA prognosis. This correlation was not evident in other types of cancer.

Aberrant sialylation is indicative of malignancy [9,35,42,43]. Previous studies have demonstrated that CD24 exhibits high sialylation levels [1,2]. However, the specific ST responsible for directly modifying CD24 is yet to be identified. Our findings revealed a significant positive association between *CD24* and *ST8SIA6*. ST8SIA6 preferentially recognizes the NeuAcα2,3(6)Gal sequence located at the non-reducing end of O-linked glycoproteins and catalyzes the formation of α2,8-disialic acid structures containing NeuAcα2,8NeuAc [44]. The NeuAcα2,8NeuAc structure catalyzed by ST8SIA6 is the ligand for murine Siglec-E, the homolog of human Siglec-7/9 [38,39,45,46,47]. Initially, in 2008, Motari et al. analyzed the glycoforms of human CD24 protein using chemical and enzymatic binding methods combined with MALDI-TOF-MS [1]. Subsequently, in 2009, Bleckmann et al. also analyzed the glycoforms of mouse CD24 protein using ESI-IT-MS and MALDI-TOF-MS techniques [2]. Notably, both investigations identified NeuAcα-2,3/6Galβ1,3GalNAc (the most abundant O-glycan), which serves as a substrate for ST8SIA6, as well as di-sialoglycans (O-glycans, potentially products of ST8SIA6) from the CD24 protein. In the present study, our in vivo and in vitro results indicate that ST8SIA6 and Siglec-E interact with CD24. Taken together, these results provide compelling evidence that CD24 undergoes modification by ST8SIA6. In BRCA, the interaction of sialylated CD24 on the tumor cell surface with Siglec-E on macrophages forms the CD24-Siglec-E axis, which enhances immunosuppression and promotes tumor growth [3]. Moreover, our correlation analysis revealed a significant positive association between Siglec-7/9 levels and macrophage infiltration in BRCA, further supporting the mechanism of the interaction of Siglec with CD24, which enhances immunosuppression.

Since its discovery, CD24 has been defined as a cell surface protein. Recently, an ever-growing number of studies have observed cytoplasmic accumulation of CD24 in samples from cancer patients. However, the impact of the subcellular location of CD24 on the clinical prognosis of cancer remains controversial. Owing to the inability of samples with immunohistochemical staining data obtained from the Human Protein Atlas database to accurately distinguish cancer grades, stage subtypes, and the corresponding number of samples, it was not possible to analyze the specific effects of the subcellular localization of CD24 on prognosis in large-scale clinical samples. We found that the α2,8-disialylation of CD24 by ST8SIA6 promoted its localization to the cell membrane. Previous research has indicated that membranous expression of CD24 significantly shortens the overall survival time in patients with BRCA [5]. Moreover, the translocation of CD24 from the intracellular compartment to the cell membrane triggers resistance treatment in BRCA [18]. Our findings further emphasize that ST8SIA6 is a key factor in promoting the membrane localization of CD24, contributing to poor prognosis and drug resistance.

## 5. Conclusions

The findings of our study revealed for the first time that the mechanism responsible for the subcellular localization of CD24 is mainly through the ST8SIA6-mediated α2,8-disialylation of CD24, promoting its localization to the cell membrane. This finding provides new insights into the functions of CD24 as a prognostic marker and innate immune checkpoint marker in BRCA. In addition, our findings establish a theoretical foundation for future investigations into the impact of CD24 localization on prognosis.

## Data Availability

All the data used during the current study are available from the corresponding author on reasonable request.

## Supplementary Materials

The following supporting information can be downloaded at: https://www.mdpi.com/article/10.3390/cells14010009/s1, Scheme S1. Immunoprecipitation analysis of the interaction between CD24 and ST8SIA6.

## Abbreviations

GPI, glycosyl-phosphatidyl-inositol-anchored; Siglecs, sialic-acid-binding immunoglobulin-like lectins; TCGA, The Cancer Genome Atlas; STs, sialyltransferases; ACC, adrenocortical carcinoma; BLCA, bladder urothelial carcinoma; BRCA, breast carcinoma; CESC, cervical and endocervical cancer; CHOL, cholangiocarcinoma; COAD, colon adenocarcinoma; DLBC, diffuse large b-cell lymphoma; ESCA, esophageal carcinoma; GBM, glioblastoma multiforme; HNSC, head and neck squamous cell carcinoma; KICH, kidney chromophobe; KIRC, kidney renal clear cell carcinoma; KIRP, kidney renal papillary cell carcinoma; LGG, lower grade glioma; LIHC, liver hepatocellular carcinoma; LUAD, lung adenocarcinoma; LUSC, lung squamous cell carcinoma; MESO, mesothelioma; OV, ovarian serous cystadenocarcinoma; PAAD, pancreatic adenocarcinoma; PCPG, pheochromocytoma and paraganglioma; PRAD, prostate adenocarcinoma; READ, rectum adenocarcinoma; SARC, sarcoma; SKCM, skin cutaneous melanoma; STAD, stomach adenocarcinoma; TGCT, testicular germ cell tumors; THCA, thyroid carcinoma; THYM, thymoma; UCEC, uterine corpus endometrial carcinoma; UCS, uterine carcinosarcoma; UVM, uveal melanoma; HR, hazard ratio; TNBC, triple-negative breast cancer; OE-*St8sia6*, *St8sia6*-overexpression; sg*St8sia6*, *St8sia6*-knockout; MFI, median fluorescence intensity; IHC, immunohistochemistry; RNA-seq, RNA sequencing; DBs, databases; TILs, tumor-infiltrating lymphocytes.
