# Supplementary material for: ST8SIA6 Sialylates CD24 to Enhance Its Membrane Localization in BRCA
Source: Cells. 2024 Dec 26;14(1):9. doi: 10.3390/cells14010009 (PMC11719756; doi:10.3390/cells14010009)
Supplement: Supplementary file 1 [file cells-14-00009-s001.zip › cells-3343129-supplementary.pdf]

Supplementary Materials for "ST8SIA6 Sialylates CD24 to Enhance Its Membrane Localization in BRCA"

Jinxia He [1, 2], Fengchao Zhang [1, 2], Baihai Wu [1, 2], Wengong Yu [1,2*]

Materials and Methods

Transient transfection

A vector containing the complete coding sequence of murine *Cd24* (pcDNA3.1-Flag-m*Cd24*) was obtained from GENEWIZ (Suzhou, China). Transient transfection was performed using Attractene Transfection Reagent (QIAGEN, Cat#301005) according to the manufacturer's instructions. On the first day, 2×10^5 4T1 cells were seeded per well in a 6-well plate. The following day, the plasmid and Attractene Transfection Reagent were separately diluted with Opti-MEM. After gently mixing the diluted plasmid and Attractene Transfection Reagent (1.2 μg of total DNA with 4.5 μL of Attractene Transfection Reagent), the mixture was incubated for 15 minutes before being added dropwise to the 4T1 cells. The medium was changed 6 hours after transfection. Twenty-four hours post-transduction, the cells were used for immunoprecipitation.

Immunoprecipitation

For the immunoprecipitation assay, cells were lysed in RIPA buffer (50 mM Tris-HCl, pH 7.4, 150 mM NaCl, 1% NP40, 0.5% sodium deoxycholate, 0.1% SDS, 1 mM EDTA, 1 mM Na3VO4, 10 mM NaF, PMSF, and Complete™ protease inhibitors). The lysates were then centrifuged at 12,000 rpm for 15 minutes at 4 °C. The cleared lysates were collected, and the protein concentration was determined using a bicinchoninic acid protein assay kit (New Cell & Molecular Biotech Co., Ltd., Cat.# WB6501). Protein A/G Magnetic Beads (Millipore, Cat.# LSKMAGAG02) were subsequently used for purification according to the manufacturer's instructions. The following antibodies were employed: anti-Flag (1 μg per sample, Sigma-Aldrich, Cat.# F1804), normal mouse IgG (1 μg per sample, Merck Millipore, Cat.# 12-371).

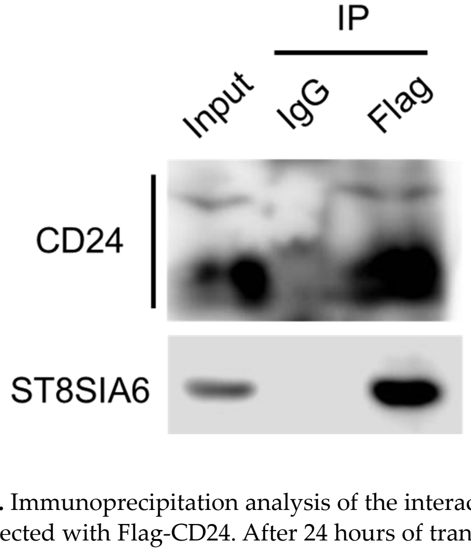

Scheme S1. Immunoprecipitation analysis of the interaction between CD24 and ST8SIA6. 4T1 cells were transfected with Flag-CD24. After 24 hours of transfection, the cellular lysates were immunoprecipitated using anti-Flag antibodies to detect the interaction between CD24 and ST8SIA6.
